# Interim analysis of a multicenter registry study of COVID-19 patients with inflammatory bowel disease in Japan (J-COSMOS)

**DOI:** 10.1007/s00535-022-01851-1

**Published:** 2022-01-28

**Authors:** Hiroshi Nakase, Yuki Hayashi, Daisuke Hirayama, Takayuki Matsumoto, Minoru Matsuura, Hideki Iijima, Katsuyoshi Matsuoka, Naoki Ohmiya, Shunji Ishihara, Fumihito Hirai, Daiki Abukawa, Tadakazu Hisamatsu, J-COSMOS group, Makoto Sasaki, Masahiro Iizuka, Mikihiro Fujiya, Takayuki Matsumoto, Fukunori Kinjo, Shiro Nakamura, Noriko Kamata, Hideki Iijima, Yuri Etani, Fumiaki Ueno, Sakiko Hiraoka, Takeo Kondo, Takashi Kagaya, Makoto Naganuma, Kiyonori Kobayashi, Taku Kobayashi, Shuji Yamamoto, Yuji Naito, Tadakazu Hisamatsu, Yoki Furuta, Keichi Mitsuyama, Yu Hashimoto, Katsuhiro Arai, Shingo Kato, Itaru Iwama, Motohiro Esaki, Hiroki Tanaka, Hiroshi Nakase, Satoshi Motoya, Atsuo Maemoto, Tomofumi Ashida, Nobuaki Nishimata, Akira Andoh, Hironori Yamamoto, Shunji Ishihara, Toshiaki Shimizu, Yasuharu Maeda, Kenji Kinoshita, Katuyuki Fukuda, Jun Kato, Ken Takeuchi, Masakazu Nagahori, Masakatsu Fukuzawa, Masayuki Saruta, Michio Itabashi, Masaru Shinozaki, Soichiro Ishihara, Naoki Yoshimura, Katsuyoshi Matsuoka, Yoichi Kakuta, Kenichi Takahashi, Ryosuke Sakemi, Sohachi Nanjo, Shusaku Yoshikawa, Keiji Ozeki, Ayako Fuchigami, Takehiko Katsurada, Kenji Watanabe, Hirotake Sakuraba, Fumihito Hirai, Takashi Hisabe, Naoki Ohmiya, Ryota Hokari, Katsuhiko Nakai, Daiki Abukawa, Shojiro Yamamoto, Kazutaka Koganei, Reiko Kunisaki, Akira Hokama

**Affiliations:** 1 Department of Gastroenterology and Hepatology, Sapporo Medical University School of Medicine, S-1, W-16, Chuoku, Sapporo, Hokkaido 060-8543 Japan; 2 Division of Gastroenterology, Department of Medicine, Iwate Medical University, Morioka, Japan; 3 Department of Gastroenterology and Hepatology, Kyorin University School of Medicine, Tokyo, Japan; 4 Department of Gastroenterology and Hepatology, Osaka University Graduate School of Medicine, Osaka, Japan; 5 Department of Gastroenterology and Hepatology, Toho University Sakura Medical Center, Chiba, Japan; 6 Department of Gastroenterology, Fujita Health University School of Medicine, Aichi, Japan; 7 Department of Gastroenterology, Faculty of Medicine, Shimane University, Izumo, Japan; 8 Department of Gastroenterology and Medicine, Fukuoka University Faculty of Medicine, Fukuoka, Japan; 9 Department of Gastroenterology and Hepatology, Miyagi Children’s Hospital, Sendai, Japan

**Keywords:** COVID-19, SARS-CoV-2, Inflammatory bowel disease, Steroid, Anti-TNF-α antibodies

## Abstract

**Background:**

The spread of coronavirus disease 2019 (COVID-19) had a major impact on the health of people worldwide. The clinical background and clinical course of inflammatory bowel disease (IBD) among Japanese patients with COVID-19 remains unclear.

**Methods:**

This study is an observational cohort of Japanese IBD patients diagnosed with COVID-19. Data on age, sex, IBD (classification, treatment, and activity), COVID-19 symptoms and severity, and treatment of COVID-19 were analyzed.

**Results:**

From 72 participating facilities in Japan, 187 patients were registered from June 2020 to October 2021. The estimated incidence of COVID19 in Japanese IBD patients was 0.61%. The majority of IBD patients with COVID-19 (73%) were in clinical remission. According to the WHO classification regarding COVID-19 severity, 93% (172/184) of IBD patients had non-severe episodes, while 7% (12/184) were severe cases including serious conditions. 90.9% (165/187) of IBD patients with COVID-19 had no change in IBD disease activity. A logistic regression analysis stepwise method revealed that older age, higher body mass index (BMI), and steroid use were independent risk factors for COVID-19 severity. Six of nine patients who had COVID-19 after vaccination were receiving anti-tumor necrosis factor (TNF)-α antibodies.

**Conclusion:**

Age, BMI and steroid use were associated with COVID-19 severity in Japanese IBD patients.

**Supplementary Information:**

The online version contains supplementary material available at 10.1007/s00535-022-01851-1.

## Introduction

The rapid spread of severe acute respiratory syndrome coronavirus-2 (SARS-CoV-2) and the resulting coronavirus disease (COVID-19) have impacted patients and healthcare workers in clinical settings [1–3]. The older group of people have a higher incidence of contracting severe COVID-19 [4] since they have weakened immune functions, which is one of the risk factors of COVID-19 [5, 6]. However, in Japan, the impact of COVID-19 on patients with various immune diseases and the factors contributing the severity of COVID-19 have yet to be elucidated.

Despite the focus on respiratory symptoms during the diagnosis of COVID-19, gastrointestinal (GI) symptoms including vomiting, diarrhea, and abdominal pain have been reported among COVID-19 patients [7–11], which indicated the involvement of SARS-CoV-2 in intestinal inflammation [12, 13].

Chronic intestinal inflammation is among the indications of inflammatory bowel disease (IBD), with an increasing prevalence rate among Japanese patients since 1950 [14]. Therefore, SARS-CoV-2 infection could affect the clinical course of IBD patients, in whom intestinal inflammation is central to the pathogenesis and who require immunosuppressive therapy.

There are differences in the severity of SARS-CoV-2 infection and the prognosis of COVID-19 patients between other countries and Japan. The genetic background of IBD is also different. Thus, it is essential to capture the clinical characteristics of Japanese IBD patients with COVID-19 for the future diagnostic and medical interventions. Therefore, we established the registry cohort of Japanese IBD patients with COVID-19 to determine the incidence of the disease and the impact of COVID-19 on the clinical course of IBD. This study was referred to as Japan COVID-19 surveillance in inflammatory bowel disease (J-COSMOS).

## Methods

This is a multicenter, registry cohort study conducted by the research study group on intractable inflammatory bowel disorder of the Ministry of Health, Labor and Welfare in Japan. The study has been conducted using information obtained from eligible patients. No new intervention was implemented for this study. The protocol of this study was approved by the IRB at each institution and registered publicly on the University Hospital Medical Information Network registration number UMIN000040656.

### Patients

Patients who were diagnosed with the following IBD types, such as (ulcerative colitis (UC), Crohn's disease (CD), Inflammatory bowel disease unclassified (IBDU), intestinal Behçet's disease (BD) and simple ulcer (SU), and who tested positive for COVID-19 were been eligible in the outpatient clinic or the participating institutions from January 1, 2020. A confirmed diagnosis of COVID-19 was defined as the presence of the SARS-CoV-2 genome as confirmed by real-time polymerase chain reaction (RT-PCR) or the positivity of the antigen test of SARS-CoV-2 via nasopharyngeal swab or saliva and the positivity of antibodies against SARS-CoV-2 in serum [15–17]. Asymptomatic SARS-CoV-2 carriers were also included. We registered IBD patients who have completed hospitalization or have undergone outpatient treatment for COVID-19 and excluded patients who have refused to participate in this study.

### Survey method

The physicians conducted the survey using the medical records at each institution and entered the obtained information into an Excel file "Case Report Form" and provided the password-locked and anonymized "Case Report Form" to the person in charge at the Department of Gastroenterology, Sapporo Medical University by e-mail. An electronic data capture (EDC) system for clinical information registration is currently used since June 2021.

### Survey items

Medical history: age, gender, height, weight, IBD diagnosis, smoking status, comorbidities (cardiovascular disease, diabetes, asthma, chronic respiratory disease, hypertension, malignancy, cerebrovascular disease, chronic renal disease, chronic liver disease, and others). Disease activity (“active” was defined as a partial Mayo score ≧ 3 with a rectal bleeding subscore ≧ 1 for UC [18, 19] and a Harvey-Bradshaw Index (HBI) ≧ 5 for CD [20], a subjective judgment of the attending physician for IBD-U, intestinal BD, and SU), duration of disease, disease type, treatment (5-aminosalicylic acid, thiopurines, steroids, calcineurin inhibitors, biologics, JAK inhibitors, nutritional therapy, cytapheresis), exacerbation of IBD, and changes in IBD treatment during COVID-19. Information on COVID-19: date of diagnosis, number of days from onset to diagnosis, testing methods that led to diagnosis (PCR, antibody, other), signs and symptoms of COVID-19 (fever, cough, dyspnea, pharyngitis, diarrhea, arthralgia-myalgia/asthenia, rhinitis, dysosmia, dysgeusia and dysphonia), presence of pneumonia, COVID-19 treatment and severity/outcomes (outpatient treatment, inpatient treatment, intensive care, death). We determined the severity of COVID-19 according to the WHO classification [21]. We defined an infection of a fully vaccinated person (at least dose of any vaccine) as a “vaccine breakthrough infection”. To examine domestic vaccination coverage over time, we calculated the vaccination coverage among the older people aged 65 years and above and among all citizens including those aged 12 years and older who were eligible for vaccination, using vaccination records published by the Cabinet Secretariat Office, based on reports from the Vaccination Record System.

### Statistical analysis

Microsoft Excel 16.0 was used to record the patient’s data and analyze the clinical background factors and results of disease. Analyses that require tests were performed using EZR software [22]. Nominal variables data were analyzed using Fisher’s exact test and the odds ratio (OR) was calculated at 95% confidence intervals (95% Cls). A t-test with 95% Cl was performed to analyze the means data. The logistic regression analysis Stepwise method (AIC, backward/forward) was performed to analyze the relationship between COVID-19 severity and risk factors. A matplot was used to describe the line plot of repeated measurement data on the IBD-activity during COVID-19.

## Results

### Patients’ characteristics

**Fig. 1:**
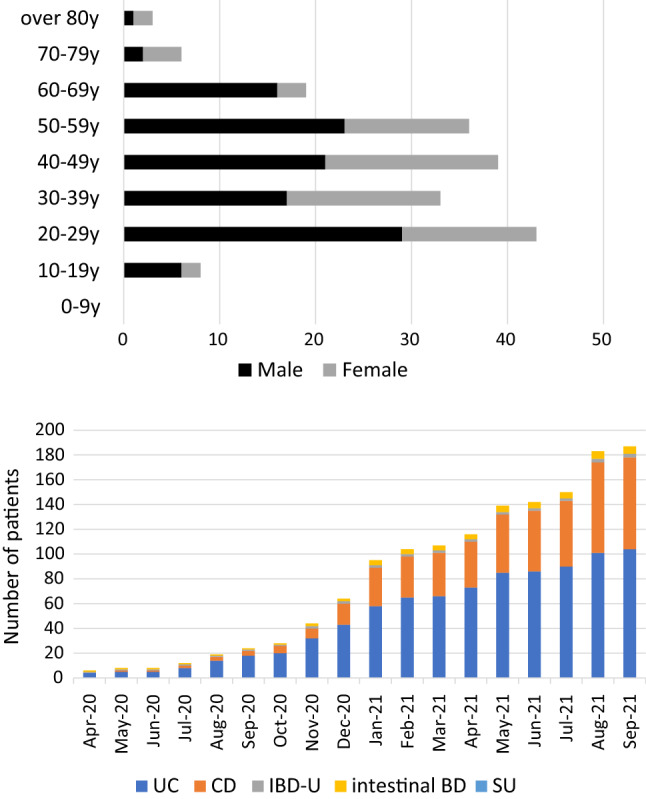
(**A**) Age distribution of Japanese IBD patients with COVID-19. (**B**) Transition of the number of Japanese IBD with COVID-19

One hundred eighty-seven IBD patients with COVID-19 were registered between June 2020 and October 2021. The mean age was 42, and the number of registered patients peaked at age 20–29 years (Fig. 1A). The diagnosis of patients was UC (*n* = 104), CD (*n* = 74), IBD-U (*n* = 3), and BD (*n* = 6). The number of females was 72 (38.5%) and 30% of registered patients had existing comorbidities. The disease activity at the diagnosis of COVID-19 was 73% (136/187) for clinical remission, 22% (42/187) for mildly active, 2.7% (5/187) for moderately active, and 2.1% (4/187) for severely active. Baseline characteristics of the patients are shown in Table 1. We calculated the total number of patients by directly asking each facility with a patient registry about the number of IBD patients attending their hospitals. Based on that the total number of patients with IBD in 72 participating facilities, which was approximately 30,500, the estimated incidence of COVID-19 in IBD patients was 0.61%.

**Table 1:** The characteristics of IBD patients with COVID-19

Patients’ profile	
Age (years) ± SD	42.0 ± 15.6
Gender (M/F) (n)	115 / 72
Height (cm) ± SD	166.0 ± 9.4
Weight (kg) ± SD	61.0 ± 12.4
BMI ± SD	21.6 ± 4.4
BMI > 30 (n)	9
Smoker (current) (%)	13/187 (7.0%)
All comorbidity (%)	58/187(31.0%)
Diagnosis of IBD	
UC	104
CD	74
IBD-U	3
Intestinal BD	6
SU	0
Disease activity (at diagnosis of COVID-19)	
Remission	136
Mild	42
Moderate	5
Severe	4

### Transition of the number of IBD patients with COVID-19 in Japan and vaccinated population

**Fig. 2:**
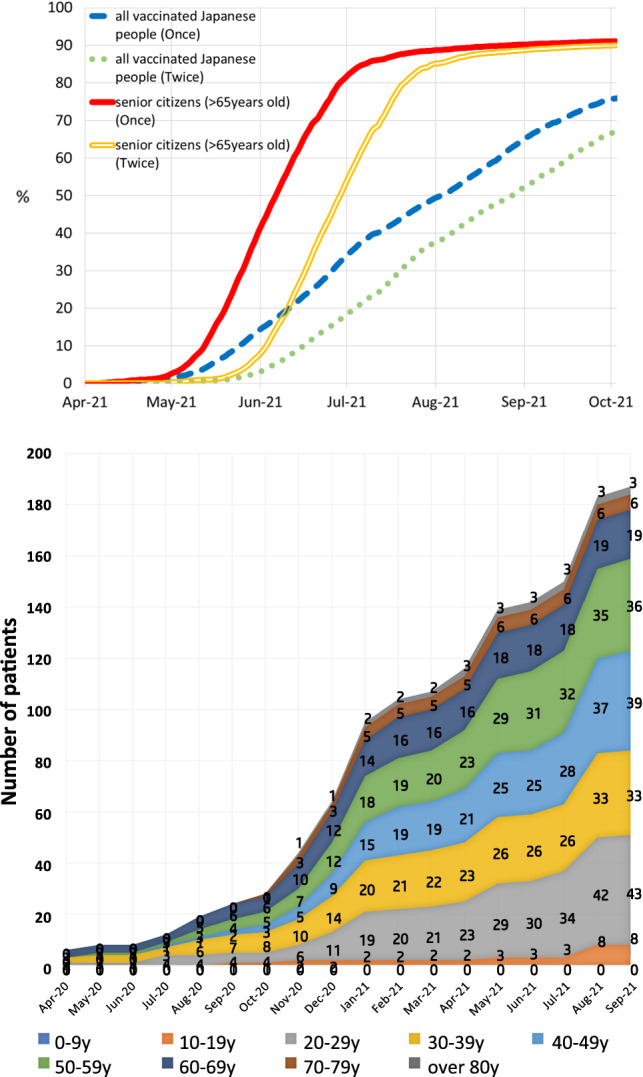
(**A**) SARS-CoV-2 vaccination coverage (%) in Japanese population. Vaccination coverage was calculated among all citizens who were eligible for vaccination, using vaccination records published by the Cabinet Secretariat Office, based on reports from the Vaccination Record System. (**B**) Transition of the number of Japanese IBD with COVID-19 stratified by age

The number of IBD patients with COVID-19 in Japan gradually increased and the upward trend was observed during the fifth wave of the pandemic (from August 2021 to September 2021) (Fig. 1B). In Japan, vaccine distribution for senior citizens started in April 2021. The first dose vaccination rate was 80% at the end of June, and the second dose vaccination rate exceeded 80% by the end of July, indicating that a large number of people have been vaccinated in a short period of time (Fig. 2A). After the start of the vaccination where the older people were prioritized, there was no significant increase in the number of registered patients over 60 years old. Meanwhile, the number of patients in the age group of 20–50 years increased (Fig. 2B). The analyses of IBD patients with COVID-19 categorized according to region in Japan showed that Kanto district had the highest number of registered patients, followed by Hokkaido and Kansai districts (Supplementary Fig. 1). This is consistent to the trend that large cities tend to have a high numbers of COVID-19 cases [23, 24].

### IBD treatment

**Fig. 3:**
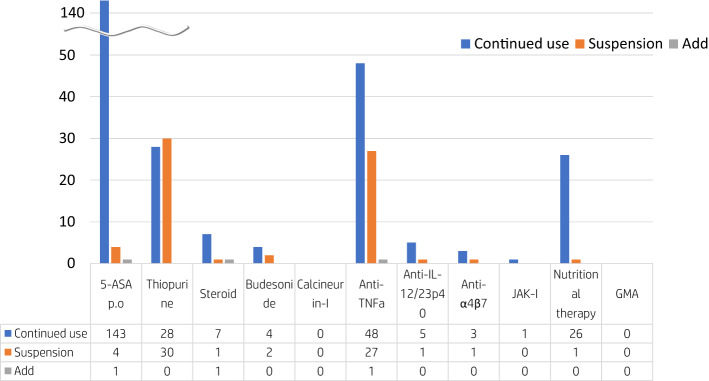
Treatment of Japanese IBD at the diagnosis of COVID-19. A blue bar indicated treatment continuation, an orange bar indicated treatment discontinuation, and a gray bar indicated additional treatment

Of the 187 registered patients, 147 were on 5-ASA, 75 on anti-TNFα antibodies, 58 on thiopurines, eight on steroids, and six on budesonide (Fig. 3). After the diagnosis of COVID-19, 52% (30/58) and 36% (27/75) of patients taking thiopurines and anti-TNFα antibodies, respectively, discontinued the medication.

### Symptoms during COVID-19

The most common COVID-19 symptoms were fever (75%), general fatigue (52%), respiratory symptoms including cough (48%), dysosmia/dysgeusia (32%/33%), and headache (24%). A total of 23% (37/160) of COVID-19 cases were related to pneumonia.

### COVD-19 severity and clinical outcome

According to the WHO classification of COVID-19 severity, 93% (172/184) of IBD patients had non-severe disease was, while 7% (12/184) of patients had severe disease. Of the 12 patients with severe COVID-19, 10 patients required oxygen and the remaining two required intensive care unit management and ventilation. No IBD patients died from COVID-19. The mean age of IBD patients with severe COVID-19 was significantly higher than that of the non-severe patients (59.3 ± 11.9 vs. 40.7 ± 15.2, p < 0.00005). In addition, the body mass index (BMI) [IQR] of IBD patients with severe COVID-19 was significantly higher than that of the non-severe patients (24.2 [23.1–29.2] vs. 21.5 [19.3–26.6], p = 0.00467). IBD patients with severe COVID-19 had significantly higher rates of any comorbidity than those with non-severe COVID-19 (OR = 7.87). Among the comorbidities, Fisher's exact test showed there are significant differences among any respiratory illness (OR = 16.3), hypertension (OR = 8.8), and cerebrovascular disease (OR = 16.3) between patients with severe COVID-19 and non-severe COVID-19 (Table 2). The rates of severe COVID-19 in patients with UC and CD were 11% (11/101) and 1.3% (1/74), respectively, although there was no statistically significant difference due to the small number of cases. In both UC and CD, neither the clinical phenotype nor disease activity was associated with COVID-19 severity. In the treatment of IBD, corticosteroids were associated with COVID-19 severity (OR = 5.37), but not budesonide (Table 3).

**Table 2:** Clinical factors associated with COVID-19 severity in Japanese IBD patients

Factor	Non-severe	Severe	Fisher-test *p*-value	Fisher-test odds
Age ± SD	40.7 ± 15.2	59.3 ± 11.9	0.00005	–
Gender M/F	107/65	7/5	0.76800	1.17
BMI [IQR]	21.5 [19.3–26.6]	24.2 [23.1–29.2]	0.00467	–
Comorbidity				
DM	4/172	1/12	0.28900	3.80
CKD	4/172	0/12	1.00000	0.00
Liver diseases	8/171	0/12	1.00000	0.00
Asthma	7/171	2/12	0.11000	4.60
COPD	0/172	0/12	–	–
Other respiratory illness	2/172	2/12	0.02180	16.30
Cardiovascular diseases	4/172	0/12	1.00000	0.00
HT	9/172	4/12	0.00551	8.80
Cerebrovascular diseases	2/172	2/12	0.02180	16.30
Malignancy	2/172	0/12	1.00000	0.00
All	47/172	9/12	0.00133	7.87
Smoking				
Never	121	6	0.11700	–
Current	12	1		
P ast smoking	29	5

**Table 3:** IBD phenotype and treatments associated with COVID-19 severity in Japanese IBD patients

Factor		Non-severe	Severe	Fisher-test *p*-value	Fisher-test odds
Diagnosis of IBD	UC	90	11	0.06480	–
	CD	73	1		
	IBD-U	3	0		
	BD	6	0		
	SU	0	0		
Clinical phenotype					
UC	Proctitis	14	1	0.93200	–
	Left side	28	4		
	Pancolitis	45	5		
	segmental	3	0		
CD	Ileitis	18	0	0.93200	–
	Colitis	15	0		
	Ileo-colitis	42	1		
	Isolated upper	14	1		
Disease activity (at diagnosis of COVID-19)	Remission	124	10	0.24600	–
	Mild	40	1		
	Moderate	5	0		
	Severe	3	1		
IBD treatment	5-ASA p.o	132/170	12/12	0.07430	Inf
	Thiopurine	55/172	2/12	0.34700	0.43
	Steroid	6/170	2/12	0.08950	5.37
	Budesonide	6/172	0/12	1.00000	0.00
	Calcineurin-inhibitors	0/172	0/12	–	–
	Anti-TNFα antibodies	73/171	1/12	0.02920	0.12
	Anti-IL-12/23p40	6/172	0/12	1.00000	0.00
	Anti-α4β7	4/172	0/12	1.00000	0.00
	JAK-inhibitor	1/170	0/12	1.00000	0.00

Missing values: three UC patients whose severity of COVID-19 according to WHO classification was not known

Changes in IBD activity before and after COVID-19 were evaluated by pMayo score in UC and by HBI in CD. We found that 90.9% (165/187) of IBD patients with COVID-19 had no change in IBD disease activity. (Supplementary Fig. 2). Regarding the association between COVID-19 severity and risk factors, a logistic regression analysis stepwise method revealed that older age (OR = 1.07), high BMI (OR = 1.18), and the steroid use (OR = 1.74) were risk factors for COVID-19, but anti-TNFα antibodies was not (Table 4, Supplementary Fig. 3, and 4).

**Table 4:** Risk factor of COVID-19 severity in Japanese IBD patients

Risk factor	Odds ratio	*p* value
Age	1.07	0.0101
BMI	1.18	0.00812
5-ASA	1.34E + 07	0.992
Steroid	1.74	0.0218

Logistic regression AIC = 66.39

Method: logistic regression analysis Stepwise method (AIC, backward/forward)

Objective variable: COVID-19 severity in the WHO classification

Explanatory variables: age, presence of comorbidities, BMI, IBD diagnosis, and IBD treatment

### COVID-19 cases after vaccination

Nine IBD patients were recorded to have acquired COVID-19 after vaccination. Five patients have CD, and four have UC, and neither had non-severe COVID-19. Three of them were considered breakthrough infections as more than 2 weeks have already passed since their second dose of vaccine. Six of the nine patients were receiving anti-TNFα antibodies, and four were receiving thiopurine. There was no progression of disease activity in nine IBD patients with COVID-19 despite the discontinuation of IBD drugs (Table 5).

**Table 5:** COVID-19 cases after vaccination

Age	Sex	Days from vaccination date (1st dose) to infection	Breakthrough cases	BMI	Diagnosis	Disease activity of IBD			Treatment of IBD			Others
						At diagnosis of COVID-19	During COVID-19	Post COVID-19	5-ASA	IM	anti-TNFα antibodies	
48	M	20	No	25.3	CD	Mild	Mild	Mild	Suspension	Suspension	Continuation	–
45	M	27	No	22.4	CD	Remission	Remission	Remission	Continuation	Suspension	Suspension	–
40	M	9	No	24.5	UC	Remission	Remission	Remission	Continuation	–	–	–
38	F	9	No	NA	CD	Remission	Remission	Remission	–	–	Suspension	–
28	M	116	Yes	19.2	UC	Remission	Remission	Remission	–	Suspension	Suspension	–
59	F	43	Yes	21.2	UC	Remission	Remission	Remission	Continuation	–	Suspension	–
64	F	26	No	12.6	CD	Remission	Remission	Remission	Continuation	–	Continuation	–
44	F	22	No	17.6	CD	Mild	Mild	Mild	Continuation	Continuation	–	IL-12/23p40 antagonist continuation
56	F	42	Yes	20.3	UC	Remission	Remission	Remission	Continuation	–	–	JAK inhibitor continuation

An infection of a fully vaccinated person (at least two injections of vaccination) is referred to as a “vaccine breakthrough infection.”

## Discussion

This is the first survey involving the characteristics and outcome of COVID-19 in IBD patients in Japan. Based on this survey, the estimated incidence of COVID-19 in patients with IBD is 0.61%, which was lower than the current incidence of COVID-19 in the general population in Japan. The incident of COVID-19 was found higher in male than in female. Fever, general fatigue, and cough were the most common COVID-19 symptoms. Regarding the disease activity and IBD medication, most of the patients diagnosed with COVID-19 were in remission and the majority had been treated with 5-ASA alone, followed by anti-TNF-α antibodies. In this current survey, the registered number of Japanese IBD patients with COVID-19 receiving steroids was low. A logistic regression analysis stepwise method revealed that older age, higher BMI, and steroid use were risk factors for COVID-19 severity. Overall, our findings confirmed that the general risk factors for severe outcomes of COVID-19 in IBD patients are similar to other studies of IBD patients.

The number of Japanese IBD patients with COVID-19 gradually increased until the fifth wave of COVID-19, however, also coincided with the largest increase of COVID-19 cases in the Japanese general population. One of the reasons for the increase in IBD patients with COVID-19 on the fifth wave is associated with the spread of SARS-CoV-2 infections in younger people, which at that time remained unvaccinated. Meanwhile, it should be noted that there was no increase in the number of older IBD patients registered during the fifth wave since the senior citizens had been priority for vaccination. The current incidence rate of COVID-19 in Japan (1.73 million/126.1 million people) is 1.37% [25]. In this cohort, the estimated incidence rate which we calculated based on the number of IBD patients registered by the participating facilities is 0.61%. This can be attributed to the fact that most of the Japanese patients with IBD often pay attention to the risk of an infection, not limited to SARS-CoV-2, and that they are particularly observing social distancing during the pandemic.

In this survey, the percentage of Japanese IBD patient with severe COVID-19 based on WHO classification was 7% and with no cases of death recorded. Surveillance Epidemiology of Coronavirus under Research Exclusion (SECURE-IBD) data showed the primary outcome (ICU/ventilation/death) was observed in 37/525 (7%) of patients, and with 3% death of reported cases [26]. Systematic review on IBD patients with COVID-19 by D’ Amico showed that 28/246 (11.4%) of patients stayed in ICU, 26/697 (3.7%) of patients needed for mechanical ventilation, and 29/760 (3.8%) of patients died [27]. These previous data are not directly comparable to our survey data in the frequency of COVID-19 severity because of the lack of severity assessment according to the WHO classification in other studies. However, it is noteworthy that there have been no deaths among Japanese IBD patient with COVID-19 up to this time. Given that about a half of patients enrolled in the cohort were on biologics and the number of patients used corticosteroids was low, the use of anti-TNFα antibodies may contribute to the lower rate of severe COVID-19, with no death cases. Several reports indicates that anti-TNFα antibodies and JAK inhibitors could regulate the pro-inflammatory cytokine production caused by COVID-19 infection [28, 29]. Further, recent clinical trial supported the suppression of COVID-19 severity by JAK inhibitors [30]. This cohort has not yet enrolled many patients on JAK inhibitors or biologics other than anti-TNFα antibodies, therefore, further survey is required.

Multivariable analysis of SECURE-IBD data demonstrated that increasing age, more than two comorbidities, systemic corticosteroid, and 5-ASA/sulfasalazine used were positively associated with adverse COVID-19 outcomes in IBD patients and the use of immunomodulatory therapy including anti-TNFα antibodies was not associated with risk of morbidity [31]. We found older age, BMI, and presence of comorbidities, and the use of corticosteroids as factors associated with increased severity, was supported by the SECURE-IBD data. Despite the small number of patients on steroids, it should be noted that steroids were still extracted as a risk factor for severe COVID-19.

In this registry, we found that the rate of severe COVID-19 in patients with UC was higher than CD. The possible reasons of the difference in the rate of severity of COVID-19 between patients with UC and CD are (1) the majority of registered patients over 60 are patients with UC (Supplementary Fig. 5) and (2) the usage rate of anti-TNFα antibodies in UC and CD patients at the diagnosis of COVID-19 was 18.2% (19/104) and 70.2% (52/74), respectively (Supplementary Table 1). These factors might contribute to the higher proportion of severe COVID-19 in UC than CD.

Regarding the change of disease activity in IBD patients with COVID-19, the results of this survey showed that COVID-19 did not affect disease activity in most IBD patients with COVID-19, except for a few IBD patients who had exacerbations. Luki and colleagues reported that COVID-19 has no durable impact of on intestinal disease of IBD patients based on change of clinical activity scores and endoscopic scores on COVID-19 positive IBD patients despite increasing the level of fecal calprotectin (fCal) at the course of COVID-19 [32]. The change of fCal without exacerbating abdominal symptoms may reflect the involvement of SARS-CoV-2 in the mild intestinal inflammation via ACE2 receptor [33], although we could not observe the change of fecal calprotectin in our survey.

Post-vaccination COVID-19 is one of the clinical challenges in COVID-19 management [34]. This survey showed nine patients had COVID-19 after first or second vaccination, three of them were considered breakthrough infections, and the others had COVID-19 before the second dose. All patients had non-severe COVID-19. Six of seven patients had received anti-TNFα antibodies and there was no worsening of disease activity in seven IBD patients with COVID-19 post vaccination despite the discontinuation of IBD drugs. A recent report indicated that anti-TNFα antibodies could affect anti-SARS-CoV-2 antibody concentrations [35, 36]. However, the association between the use of anti-TNFα antibodies and the onset of COVID-19 after vaccination remains unclear in the current survey because we could not measure antibodies against SARS-CoV-2 at this time. A study on antibody titer after COVID-19 vaccination in Japanese IBD patients is underway, and the result would illuminate how IBD drugs affect SARS-CoV-2 antibody concentrations after vaccination.

Our study has several limitations. First, not all IBD patients with COVID-19 in Japan were included because of lack of national registry for IBD patients including pediatric patients. However, the data is reliable because all patients were registered from facilities with IBD specialists. Second, the incidence of COVID-19 after two doses of vaccination has not yet been determined, and it is important to continue the registry in the future. Third, in the present survey, we did not collect data regarding the types of anti-TNF-α antibody agent in detail. The rate of discontinuation of anti-TNF-α antibody agents was high after patients had COVID-19; however, we should consider that differences in administration intervals may have influenced the decision of the discontinuation of the agents.

In conclusion, we first reported the results of the interim analysis of the COVID-19 registry in Japanese IBD patients. The incidence of COVID-19 in Japanese IBD patients was found to be lower than the general population. Older age and the presence of comorbidities were factors in the incidence of contacting COVID-19, which was the same trend as in the general population. Various factors related to the risk and severity of COVID-19 may be extracted based on accumulation of COVID-19 cases in the future. Further, follow-up study is necessary to confirm our current results.

## Supplementary Information

Below is the link to the electronic supplementary material.

Supplementary Fig.1. The number of IBD patients with COVID-19 by region in Japan (DOCX 370 kb)

Supplementary Fig.2. Changes in IBD activity during COVID-19. The line plot of repeated measurement data for analyzing the IBD disease activity during COVID-19 showed the course of all severe and non-severe patients. The mean pMayo or HBI score was calculated for each time axis (DOCX 221 kb)

Supplementary Fig.3. The age distribution of patients with COVID-19 and the COVID-19 severity rate (DOCX 20 kb)

Supplementary Fig.4. The BMI distribution of patients with COVID-19 and the COVID-19 severity rate. (DOCX 17 kb)

Supplementary Fig.5. Age distribution of UC and CD patients with COVID-19 (DOCX 392 kb)

Supplementary file6 (PPTX 40 kb)
