# Supplementary material for: Interim analysis of a multicenter registry study of COVID-19 patients with inflammatory bowel disease in Japan (J-COSMOS)
Source: J Gastroenterol. 2022 Jan 28;57(3):174–84. doi: 10.1007/s00535-022-01851-1 (PMC8795939; doi:10.1007/s00535-022-01851-1)
Supplement: Supplementary file 2 — Supplementary Fig.2. Changes in IBD activity during COVID-19. The line plot of repeated measurement data for analyzing the IBD disease activity during COVID-19 showed the course of all severe and non-severe patients. The mean pMayo or HBI score was calculated for each time axis (DOCX 221 kb) [file 535_2022_1851_MOESM2_ESM.docx]

Supplement Figure 2. Changes in IBD activity during COVID-19.

pMayo

0

2

4

6

8

10

Non severe

Severe

After cure of COVID-19

During COVID-19

At diagnosed

of COVID-19

WHO classification severity of COVID-19

HBI

0

2

4

6

8

Non severe

Severe

WHO classification severity of COVID-19

At diagnosed

of COVID-19

During COVID-19

After cure of COVID-19
