# Supplementary material for: Interim analysis of a multicenter registry study of COVID-19 patients with inflammatory bowel disease in Japan (J-COSMOS)
Source: J Gastroenterol. 2022 Jan 28;57(3):174–84. doi: 10.1007/s00535-022-01851-1 (PMC8795939; doi:10.1007/s00535-022-01851-1)
Supplement: Supplementary file 4 — Supplementary Fig.4. The BMI distribution of patients with COVID-19 and the COVID-19 severity rate. (DOCX 17 kb) [file 535_2022_1851_MOESM4_ESM.docx]

**Supplementary Figure 4**
