# Supplementary material for: Interim analysis of a multicenter registry study of COVID-19 patients with inflammatory bowel disease in Japan (J-COSMOS)
Source: J Gastroenterol. 2022 Jan 28;57(3):174–84. doi: 10.1007/s00535-022-01851-1 (PMC8795939; doi:10.1007/s00535-022-01851-1)
Supplement: Supplementary file 6 — Supplementary file6 (PPTX 40 kb) [file 535_2022_1851_MOESM6_ESM.pptx]

## Slide 1
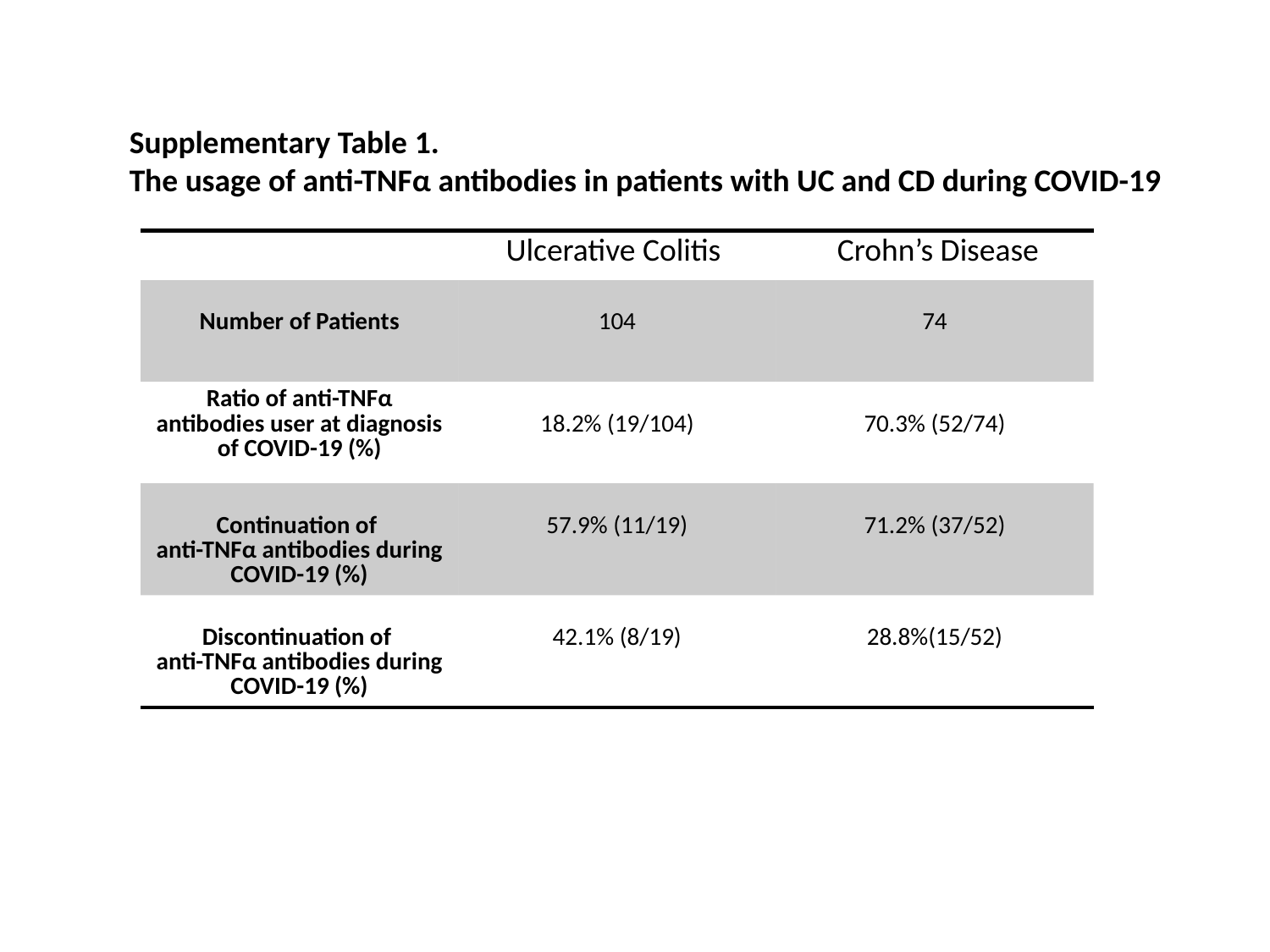

Supplementary Table 1.
The usage of anti-TNFα antibodies in patients with UC and CD during COVID-19
| | Ulcerative Colitis | Crohn’s Disease |
| --- | --- | --- |
| Number of Patients | 104 | 74 |
| Ratio of anti-TNFα antibodies user at diagnosis of COVID-19 (%) | 18.2% (19/104) | 70.3% (52/74) |
| Continuation of anti-TNFα antibodies during COVID-19 (%) | 57.9% (11/19) | 71.2% (37/52) |
| Discontinuation of anti-TNFα antibodies during COVID-19 (%) | 42.1% (8/19) | 28.8%(15/52) |
