# Supplementary material for: Interim analysis of a multicenter registry study of COVID-19 patients with inflammatory bowel disease in Japan (J-COSMOS)
Source: J Gastroenterol. 2022 Jan 28;57(3):174–84. doi: 10.1007/s00535-022-01851-1 (PMC8795939; doi:10.1007/s00535-022-01851-1)
Supplement: Supplementary file 4 [file 535_2022_1851_MOESM1_ESM.docx]

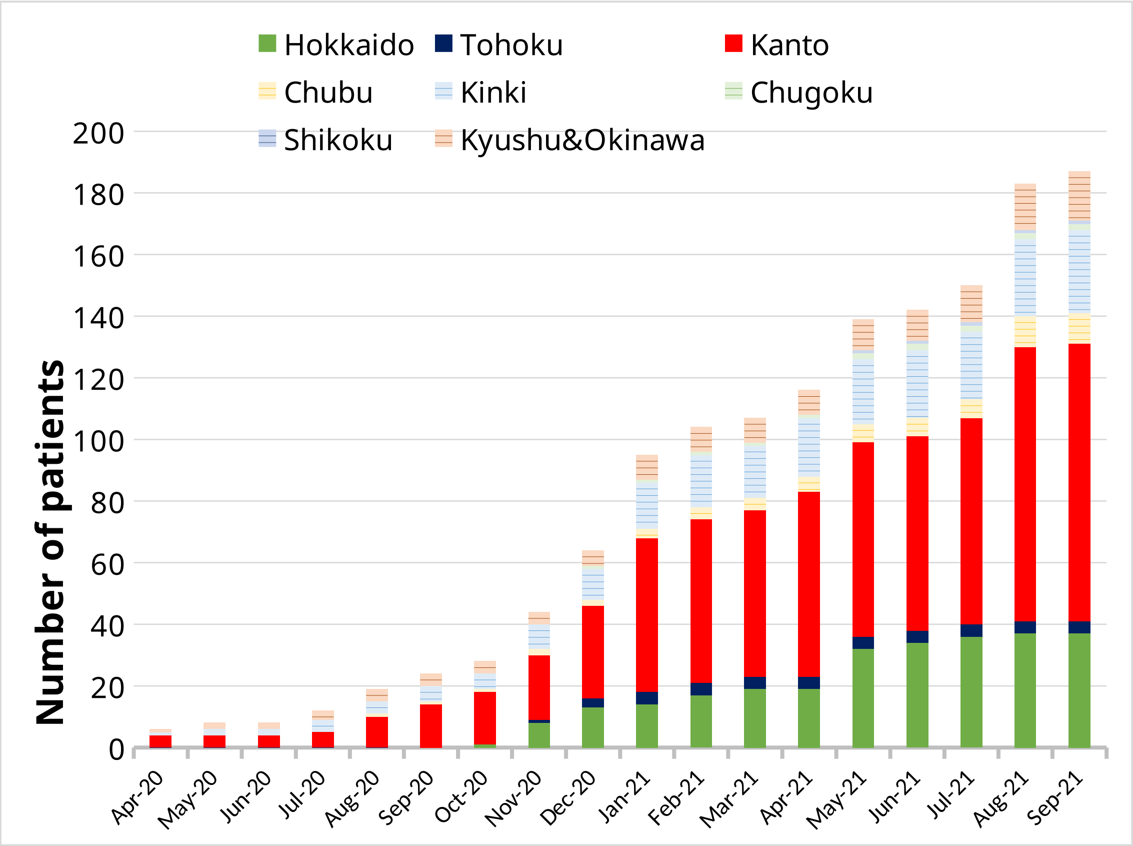
Hokkaido
Tohoku
Kanto
Chubu
Kinki
Chugoku
Shikoku
Kyushu&Okinawa
Number of patients
200
180
160
140
120
100
80
60
40
20
0
Apr-20
May-20
Jun-20
Jul-20
Aug-20
Sep-20
Oct-20
Nov-20
Dec-20
Jan-21
Feb-21
Mar-21
Apr-21
May-21
Jun-21
Jul-21
Aug-21
Sep-21
